# Efficient isolation of human gingival stem cells in a new serum-free medium supplemented with platelet lysate and growth hormone for osteogenic differentiation enhancement

**DOI:** 10.1186/s13287-022-02790-7

**Published:** 2022-03-25

**Authors:** Ihsène Taihi, Caroline Pilon, José Cohen, Ariane Berdal, Bruno Gogly, Ali Nassif, Benjamin Philippe Fournier

**Affiliations:** 1 Laboratory of Molecular Oral Pathophysiologie, Centre de Recherche des Cordeliers, INSERM, Université de Paris, Sorbonne Université, 75006 Paris, France; 2 AP-HP, site hospitalier Charles Foix-Pitié Salpêtrière, 94200 Ivry, France; 3 AP-HP, site hospitalier Henri Mondor, CIC-BT-504, INSERM UMRS 955, Paris-Est University, Créteil, France; 4 AP-HP, sites hospitaliers Pitié Salpêtrière et Rothschild, Département d’Orthopédie Dento-Faciale, Centre de Référence Maladies Rares Orales et Dentaires (O-Rares), 75013-75019 Paris, France

**Keywords:** Mesenchymal stem cell, Gingiva, Neural crest, Culture medium, Serum free, Growth hormone, Bone regeneration, Immunomodulation

## Abstract

**Background:**

The use of distant autografts to restore maxillary bone defects is clinically challenging and has unpredictable outcomes. This variation may be explained by the embryonic origin of long bone donor sites, which are derived from mesoderm, whereas maxillary bones derive from neural crest. Gingival stem cells share the same embryonic origin as maxillary bones. Their stemness potential and ease of access have been repeatedly shown. One limitation in human cell therapy is the use of foetal calf serum during cell isolation and culture. To overcome this problem, a new serum-free medium enriched with an alternative to foetal calf serum, i.e., platelet lysate, needs to be adapted to clinical grade protocols.

**Methods:**

Different serum-free media enriched with platelet lysate at various concentrations and supplemented with different growth factors were developed and compared to media containing foetal calf serum. Phenotypic markers, spontaneous DNA damage, and stem cell properties of gingival stem cells isolated in platelet lysate or in foetal calf serum were also compared, as were the immunomodulatory properties of the cells by co-culturing them with activated peripheral blood monocellular cells. T-cell proliferation and phenotype were also assessed by flow cytometry using cell proliferation dye and specific surface markers. Data were analysed with t-test for two-group comparisons, one-way ANOVA for multigroup comparisons and two-way ANOVA for repeated measures and multigroup comparisons.

**Results:**

Serum-free medium enriched with 10% platelet lysate and growth hormone yielded the highest expansion rate. Gingival stem cell isolation and thawing under these conditions were successful, and no significant DNA lesions were detected. Phenotypic markers of mesenchymal stem cells and differentiation capacities were conserved. Gingival stem cells isolated in this new serum-free medium showed higher osteogenic differentiation potential compared to cells isolated in foetal calf serum. The proportion of regulatory T cells obtained by co-culturing gingival stem cells with activated peripheral blood monocellular cells was similar between the two types of media.

**Conclusions:**

This new serum-free medium is well suited for gingival stem cell isolation and proliferation, enhances osteogenic capacity and maintains immunomodulatory properties. It may allow the use of gingival stem cells in human cell therapy for bone regeneration in accordance with good manufacturing practice guidelines.

**Supplementary Information:**

The online version contains supplementary material available at 10.1186/s13287-022-02790-7.

## Background

Mesenchymal stem cells (MSCs), first identified by Friedenstein in bone marrow (BM-MSCs), are used in regenerative medicine to treat several diseases [18]. Interest in applying MSCs to cell therapy is growing due to their stemness properties like self-renewal, multipotency, clone formation and immunomodulatory capacity [50]. Many sources of human MSCs are available in addition to BM — the cells can be isolated from adipose tissue, skin, and connective tissue of the oral cavity. A particular advantage of MSCs in human cell therapy is that they present no ethical issues, unlike embryonic stem cells. Moreover, they have low immunogenicity and can be used in treating cancer and many systemic diseases [11, 39].

In the craniofacial region, regeneration of jawbone defects presents a real challenge for oral and maxilla-facial surgeons. Regardless of aetiology (e.g., periodontal disease, alveolar bone resorption after tooth loss, benign or malignant tumours, congenital or traumatic lesions), restoration of jawbone defects is necessary to restore oral functions such as chewing, deglutition, phonation, and facial aesthetic.

Autografts derived from tibia or iliac bones are currently the gold standard for surgical treatments, but the need to create a second surgical (i.e., donor) site and the high risk of morbidity at that site are still major drawbacks. The clinical outcomes for bone regeneration treatments remain inconsistent and controversial, but may be explained by differences in the embryonic origin, cellular phenotypes or tissue microenvironment of the donor site and acceptor site [37].

Maxillary and mandible bones are derived from neural crest, while appendicular bones derive from paraxial and lateral mesoderm [27, 33]. Regenerating bone with stem cells of the same embryonic origin might offer a superior alternative to restore jawbone defects.

Neural crest-derived MSCs from the craniofacial region and especially from the gingiva have been thoroughly studied. Gingiva is mainly composed of gingival fibroblasts (GFs), which are active in producing extracellular matrix and collagen fibres and give the high healing potential and force-bearing characteristics of this tissue [26]. Furthermore, neural crest-derived MSCs can have immunomodulatory effects and contain subpopulations of stem cells.

Stem cells derived from gingiva (GSCs) have been thoroughly studied within the past ten years, and seem advantageous for human cell therapies [14]. They can be harvested easily and non-invasively, with no scar formation or functional problems when compared to BM-MSCs, adipose-derived MSCs (AD-MSCs) or dental pulp stem cells, which require prior tooth removal. The multipotency and immunomodulatory properties of GSCs have already been investigated in different studies [9, 15, 16, 47, 54]. Indeed, they offer a promising tool in regenerative medicine, especially for maxillo-facial bone regeneration.

To achieve successful cell-based bone therapy to compensate bone defects, sufficient stem cells or osteogenic progenitors are needed. For this, cells must be isolated, expanded in vitro, and loaded on transferring material before they can be implanted at a specific anatomical site for treatment of the bone defect. To grow and expand a sufficient number of MSCs, culture media are classically supplemented with foetal calf serum (FCS), which supports cell adhesion, expansion and differentiation [4]. However, the use of such xenogeneic supplements involves risks of immunogenic reactions and transmission of zoonotic diseases [6, 24, 46]. This has led many researchers in the last few years to propose alternative sources to FCS and develop serum-free culture media (SFM) to improve the safety of MSC transplantation and be compatible with human cell-based therapy requirements [8, 19, 23]. Platelet lysate (PL) is one of the main alternatives to FCS and has been widely studied.

Indeed, PL contains a large array of growth factors and cytokines. This alternative to FCS may minimise immunological reactions and allow in vitro culture of MSCs without compromising their functional and biological properties [31, 35]. Such advantages and attributes should be available to other cell types and, more precisely, to GSCs to achieve reliable and safe expansion and differentiation of cells for GSC-based bone regeneration.

While many studies have confirmed the capacity of PL to support BM-MSC and AD-MSC proliferation [10, 21], few have characterised the processes of isolation, expansion and proliferation of these cells and most importantly their immunomodulatory properties in SFM supplemented with PL (SFM-PL). Indeed, most studies have used FCS culture media for primary isolation and cryopreservation of GSCs [43]. Moreover, osteogenic differentiation in SFM has rarely been studied.

Therefore, the main purpose of our study was to define the most appropriate SFM supported with PL as an alternative to FCS. To do this, we first compared FCS and PL components and added some essential factors present in FCS to our SFM-PL and used it for isolation, expansion and osteogenic differentiation of human GSCs. Second, we assessed the effects of this new SFM-PL on cellular phenotypes, stemness properties, differentiation potential and immunomodulation.

## Materials and methods

### Conception of SFM and validation of an ideal medium for proliferation

#### Gingival stem cell isolation

Gingival tissue was collected from four donors during surgical tooth removal after their informed consent. All isolation protocols conformed with the Helsinki Declaration 1994 and were approved by the local ethics committee. GSC isolation was performed as previously described [17].

#### Proliferation medium preparation

Serum-free basic medium was composed of Dulbecco’s Modified Eagle Medium Low Glucose (DMEM-LG), GlutaMAX and pyruvate supplement, 5 IU/mL Penicillin–Streptomycin (Gibco), 1% non-essential amino acids (NEAA; Gibco), 2.5 mg/L of Amphotericin B (250 µg/mL; Gibco), and l-ascorbic acid 2-phosphate (50 µg/mL; Sigma-Aldrich). Heparin 2 IU/ml (2000 IU, Tebu-Bio) was added to prevent gel formation under PL conditions.

GSC proliferation (*n* = 4) was tested using five different SFM: Dulbecco’s Modified Eagle Medium Low Glucose (DMEM-LG) supplemented with PL (Tebu-Bio, Offenbach, Germany) at 1%, 2.5%, 5% and 10% or SF Dulbecco’s Modified Eagle Medium F12 (DMEM-F12). After selecting the best PL%, several different growth factors were added individually: recombinant human growth hormone (GH) at 4 ng/mL (Peprotech, USA), testosterone at 10 pg/mL (Sigma-Aldrich), recombinant human epidermal growth factor (EGF) at 10 ng/mL (Peprotech, USA), transforming growth factor-beta 1 (TGFβ1) at 10 ng/mL (Peprotech, USA), recombinant human basic fibroblast growth factor (bFGF) at 10 ng/mL (Peprotech, USA). These different SFM were compared to medium containing 10% FCS.

#### Cell proliferation assay

**Fig. 1:**
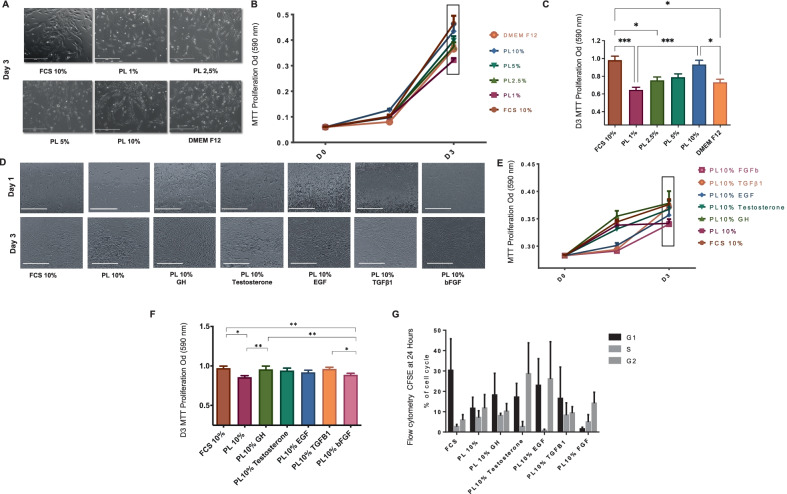
Validation of PL10% + GH as an ideal SFM for GSC proliferation. **A** Microscopic views at 24 h and 72 h of GSCs (*n* = 4) cultured in PL 1%, 2.5%, 5% and 10% and compared to FCS10% and DEMEM F12 SFM. **B** and **C** MTT growth assay at 24 h and 72 h in FCS10%; PL 1%, 2.5%, 5%, 10% and DMEM F12. PL10% medium yielded the highest normalized proliferation rate among SFM for GSCs. **D** Microscopic views at 24 h and 72 h of GSCs (*n* = 4) cultured in PL10% and different human supplements: GH, testosterone, EGF, TGFβ1 and bFGF and compared to FCS10% medium. **E** and **F** MTT growth assay at 24 h and 72 h in PL10% and different human supplements: GH, testosterone, EGF, TGFβ1 and bFGF and compared to FCS10% medium. PL10% + GH medium yielded the highest rate of GSC proliferation among SFM when compared to PL10% alone. **G** Cell cycle analysis with CFSE at 24 h of GSC culture in PL 10% and the following single supplements: GH, testosterone, EGF, TGFβ1 and bFGF and compared to FCS10%. Both GH and TGFβ1 increased the proportion of GSCs in S-phase. Scale bars: **A** 400 µM, **D** 400 µM

The cell proliferation assay aimed to identify the optimal concentration of PL and the ideal supplement. First, the effect of a gradual increase of PL concentration from 1 to 10% (PL1%, PL2.5%, PL5%, PL10%) was assessed on GSC expansion and compared to DMEM F12 and FCS 10% media. Cells were seeded into 24-well plates (Falcon-BD) in triplicates at 5000 cells/well in 500 µL of each condition at 37 °C in a humidified, 5% CO_2_ incubator. At 24 h and 72 h, 50 µL of 3-(4,5-dimethylthiazol-2-yl)-2,5-diphenyltetrazolium bromide (MTT, 5 mg/mL in phosphate-buffered saline (PBS); Sigma) was added to each well and subsequently incubated at 37 °C for 4 h. 500 µL of dimethyl sulfoxide (DMSO) (Thermo Fisher Scientific) was added to each well and mixed for 30 min on a shaker. The absorbance at 490 nm was then measured using a 96-well flat-bottomed microtitre plate reader. Each sample was assayed in triplicate and each well was read in triplicate. Cells incubated with FCS 10% culture medium were used as a control group. Based on the results of this assay, we chose PL10% as the optimal SFM (Fig. 1A–C). Then, the cells were harvested under the following conditions: FCS10%, PL10%, PL10% + GH, PL10% + testosterone, PL10% + EGF, PL10% + TGFβ1 and PL10% + bFGF. The MTT proliferation assay was performed at 24 h and 72 h as described above to define the ideal supplement to PL media.

#### Cell cycle analysis using flow cytometry

Flow cytometric analysis of the cell-cycle distribution of GSCs (*n* = 4) was performed for the following conditions: FCS10%, PL10%, PL10% + GH, PL10% + testosterone, PL10% + EGF, PL10% + TGFβ1 and PL10% + bFGF. For this, 10^6^ cells were initially harvested after Trypsin–EDTA treatment and centrifugation. Cells were treated with carboxyfluorescein succinimidyl ester (CFSE) (10^−5^ mM) (CellTrace CFSE Cell Proliferation Kit, Thermo Fisher Scientific) in 10 mL of FCS10% medium for 10 min at 37 °C. 10^5^ cells were not treated and served as a control. Cells were washed twice by pipetting with preheated PBS (1X; Thermo Fisher Scientific) after centrifugation. The treated cells were seeded into 6-well plates (Falcon-BD) in duplicates at 50,000 cells/well in 3 mL of each culture condition at 37 °C in a humidified, 5% CO2 incubator. After 24 h, the cells were harvested and put in a filtered solution of PBS/BSA 2% (bovine serum albumin, Sigma-Aldrich). Samples were analysed in a BD LSR II flow cytometer (BD Biosciences). The absorbance of CFSE was measured at 488 nm and the cell cycle was characterised by flow cytometry using FlowJo Software v10.8 (BD Life Sciences) (flowjo.com).

### Cell isolation in the new SFM, proliferation and cell characterisation

#### L-GSC isolation in the new SFM

In a second set of experiments, GSCs were isolated in the new selected SFM. Gingival tissue was collected from three donors during surgical tooth removal after their informed consent. The same protocols were followed to obtain cells with no FCS contamination, which were designated as L-GSCs, in contrast to S-GSCs, which were isolated from medium containing FCS 10%.

#### Immunophenotypic analysis

Surface protein expression of newly isolated L-GSCs (*n* = 3) at passage 3 was analysed and compared to that of S-GSCs (*n* = 3) with flow cytometry (see Additional file 1: Material and methods). Data were analysed using FlowJo Software v10.8 (BD Life Sciences) (flowjo.com).

#### Immunofluorescence

L-GSCs and S-GSCs were grown on glass coverslips and fixed. Cells were stained with primary antibodies and corresponding fluorescent-tagged secondary antibodies, counterstained with DAPI and mounted in G-mount medium (Invitrogen). Images were collected using a Zeiss Axiovert 200 M fluorescence microscope.

#### DNA damage

To verify the absence of DNA lesions within the gingival stem cells isolated from the selected SFM, L-GSCs (*n* = 3) and S-GSCs (*n* = 3) were tested using an HCS DNA Damage Kit (H.10292, Invitrogen) according to the manufacturer’s instructions. The positive control was treated with Menadione (3.7 µMol/L) (M57405, Sigma-Aldrich) for 1 h at 37 °C [2] (see Additional file 1: Material and methods.)

#### RT-qPCR

The investigated genes for undifferentiated GSCs were *GHRU, GHR1* and *TGFβR1*. *SDHA and ACTB* were used as reference genes [47] to normalise the relative expression of the target genes (Table 1). For osteogenic differentiation, *ALP, DSPP, OCN and OPN* were used as target genes. *GAPDH* and *SDHA* were used as reference genes. For adipogenic differentiation, *PPAR γ* and *LPL* were used as target genes. *GAPDH* was used as a reference gene. For this, L-GSCs and S-GSCs were amplified in their respective media and then harvested after Trypsin–EDTA treatment and centrifugation. The cells were frozen at − 80 °C and stored until used for RNA extraction. Total RNA was isolated (GSC *n* = 3) using a ReliaPrep RNA Cell Miniprep System kit, (Promega) according to the manufacturer’s instructions. RNA concentrations and purity were assessed by a Nanodrop spectrophotometer (Thermo Scientific, Wilmington, DE, USA). 2 μg of RNA from each sample was reverse-transcribed using SuperScript II Reverse Transcriptase (Invitrogen) as described in the provided protocol. RT-qPCR were performed as previously described [47].

**Table 1:** Primers sequences (target genes and housekeeping genes)

Primer	Forward primers (5′–3′)	Reverse primers (5′–3′)
ALP	CGT-GGC-TAA-GAA-TGT-CAT-CAT-GTT	GAT-TTC-CCA-GCG-TCC-TTG-GC
DSPP	CAA-CCA-TAG-AGA-AAG-CAA-ACG-CG	TTT-CTG-TTG-CCA-CTG-CTG-GGA-C
GAPDH	GAC-CCC-TTC-ATT-GAC-CTC-AAC-TAC	AAG-TTG-TCA-TGG-ATG-ACC-TTG-GCC
GH-R1	GAT-CAG-AGG-CGA-AGC-TCG-GA	AGC-ATC-ACT-TGA-TCC-TGC-CA
GH-RU	CCA-TTG-CCC-TCA-ACT-GGA-CTT	AAT-ATC-TGC-ATT-GCG-TGG-TGC
LPL	CAG-GAT-GTG-GCC-CGG-TTT-AT	CAG-GAT-GTG-GCC-CGG-TTT-AT
OCN	CTT-GGT-GCA-CAC-CTA-GCA-GA	ACC-TTA-TTG-CCC-TCC-TGC-TT
OPN	AGC-CAG-GAC-TCC-ATT-GAC-TCG-AAC	GTT-TCA-GCA-CTC-TGG-TCA-TCC-AGC
PPAR	GCG-ATT-CCT-TCA-CTG-ATA-C	CTT-CCA-TTA-CGG-AGA-GAT-CC
SDHA	AGC AAG CTC TAT GGA GAC CT	TAA TCG TAC TCA TCA ATC CG
TGFß-R1	TGG-TAC-CCA-AGG-AAA-GCC-AG	CCA-CTC-TGT-GGT-TTG-GAG-CA
UBC	GTG GCA CAG CTA GTT CCG T	CTT CAC GAA GAT CTG CAT TGT CA

#### CFU-F assay

Colony-forming capacity was determined for L-GSCs and compared to that of S-GSCs. For this, 1500 cells were seeded in 100-mm Petri dishes in 10 mL of medium for each condition. After 10 days of culture, cell colonies were stained with crystal violet. Colony counting was performed using ImageJ software.

### Multipotent differentiation of GSCs in the new SFM

The capacity of GSCs to differentiate into osteogenic, adipogenic, myofibroblast and neural precursors was carried out at passage three (*p* = 3) for both S-GSCs and L-GSCs.

### Osteogenic differentiation

The osteogenic medium consisted of DMEM-LG supplemented with 20% FCS, 10 ml/L Penicillin–Streptomycin (5 IU/ml), 1% NEAA, 2.5 mg/L Amphotericin B (250 μg/ml), 50 μg/ml l-ascorbic acid 2-phosphate, 100 nM dexamethasone, and 10 mM β-glycerophosphate (FCS osteogenic medium) [47].

The effect of increasing concentrations of PL from 1 to 20% (PL1%, PL2.5%, PL5%, PL10% and PL20%) was assessed on GSC differentiation. SFM were also supplemented with heparin 0.6 IU/mL to prevent gel formation (2000 IU, Tebu-Bio) [25]. For this experiment, GSCs (*n* = 2) and ASCs (*n* = 6) (Adipose-derived stem cells, ZenBio company, USA) at early passages (1–5) were seeded in 6-well plates (5 × 10^5^ cells per well) in 2 mL of each medium of the six osteogenic conditions (5 SF, 1 FCS). Fresh media were added every 72 h. Dexamethasone was supplemented every 7 days. After 28 days, cell viability was assessed by Calcein AM staining. Cells were then fixed in paraformaldehyde (PFA) 4%/sucrose 5% solution and stained with Alizarin Red S [47]. The spectrometry of Alizarin Red S allowed us to measure the calcium content, as previously described [47].

### Fluorescent dyes for mineralisation examination

Xylenol orange powder (Sigma, St. Louis, MO) was dissolved in distilled water and filtered to make the 20 mM stock solution. Xylenol orange was added overnight to each plate at day 15 of osteogenic differentiation, to a final concentration of 10% of the final medium (v/v). Fresh medium without fluorochrome was added to the plates to avoid non-specific fluorescent background before microscopic and photographic analyses. A TRITC Red filter was used to reveal xylenol orange staining by fluorescent microscopy [51].

### Scanning electronic microscopy

Cells were seeded on a cancellous particulate allograft (ZIMMER-BIOMET) in either FCS20% or PL5% osteogenic medium for 21 days in suspension with the droplet technique. For SEM analysis, cells and scaffolds were fixed with PIPES buffer/PFA4%, treated with 0.5% osmium tetroxide to increase the contrast and dehydrated in specific grade alcohol. Samples were coated with gold by standard protocols and examined using a scanning electron microscope at 400 × magnification.

### Adipogenic differentiation

The adipogenic medium contained Dulbecco’s Modified Eagle Medium High Glucose (DMEM -HG) 4.5 g/L supplemented with FCS 10%, 1 µM dexamethasone, 5µg/ml insulin, and 1mM of  3-isobutyl-1-methylxanthine. Oil Red O staining was performed, and the spectrometric measurements recorded (see Additional file 1: material and methods).

### Myofibroblast differentiation

For myofibroblast induction, L-GSCs and S-GSCs were cultured in DMEM supplemented with 3% FCS and 10 ng/mL TGF-β1 (US Biological) for 7 days. Immunocytochemistry of α-SMA and actin was performed on differentiated cells as described above (Immunofluorescence section).

### Neurosphere formation

For neurosphere formation, 4 × 10^5^ cells were cultured in 35-mm low-attachment culture dishes (Falcon) for 7 days in neurosphere-forming medium composed of Nutrient Mixture F-12 (DMEM/F12) (GIBCO) supplied with B-27 Supplement 50× (ThermoFisher Scientific), bFGF 20 ng/mL and EGF 10 ng/mL (Peprotech) [16]. The formed spheres were then transferred to 24-well plates and imaged with an inverted microscope (EVOS Digital Microscopes). Sphere number and size were measured using ImageJ software (http://rsb.info.nih.gov/ij/). Spheres were then stained with Nestin (1:100, DSHB) and β-III tubulin as described above (Immunofluorescence section).

### Immunomodulatory capacity of L-GSCs

#### Flow cytometric analysis of activated PBMCs in the presence of GSCs

To study the influence of isolating GSCs in SFM, the immunomodulatory capacity of L-GSCs was compared to that of S-GSCs. Cocultures were performed with activated human PBMCs.

PBMCs were cocultured with L-GSCs (*n* = 3) or S-GSCs (*n* = 3) in their respective media. For each type of GSC, there were 4 culture conditions: (i) non-activated PBMCs (NA), (ii) PBMCs (A) activated with Dyna-beads Human T-Activator CD3/CD28 (Cat. No. 111.31D, Invitrogen), (iii) PBMCs with GSCs (NA + G), and (iv) activated PBMCs with GSCs (A + G).

Briefly, GSCs were seeded in a 48-well plate (50,000 cell/plate) in NA + G and A + G media and put in a humid 37 °C incubator with 5% CO_2_ for 1 h. Meanwhile, PBMCs were treated with Cell Proliferation Dye (CPD) eFluor 450 (Cat. No. 65-0842-85, eBioscience) diluted (1:1000) in a PBS/FCS 3% solution for 10 min at 37 °C in the dark. FCS medium was added to PBMCs, and the cells were centrifugated at 1600 RPM for 5 min. PBMCs were seeded in the 48-well plate (200,000 cells/plate) in either FCS or PL proliferation medium. Anti-CD3/CD28 beads were washed twice in either FCS or PL medium before seeding in A and A + G condition plates (200,000 beads/plate) (Ratio: 1GSC: 4PBMC and 1PBMC: 1 bead). After 72 h, PBMCs were harvested in PBS/FCS 3% solution and distributed in triplicates in a 96-well plate treated with three mixtures of antibodies corresponding to different T-cell surface markers for 30 min at 4 °C.

Cells were then washed twice and transferred into polystyrene round-bottomed tubes (BD Biosciences) for cell analysis with a BD LSR II flow cytometer (BD Biosciences). Data were analysed using FlowJo Software v10.8 (BD Life Sciences) (flowjo.com).

### Statistical analysis

Data are presented as mean ± standard error (SEM) and were analysed with t-test for two-group comparisons, one-way ANOVA for multigroup comparisons and two-way ANOVA for repeated measures and multigroup comparisons. Data were statistically significant if *p* < 0.05.

## Results

### Validation of the ideal SFM for GSC proliferation and osteogenic differentiation

#### SFM supplemented with PL 10% and GH is optimal for GSC proliferation

To define the optimal SF culture medium to isolate and amplify GSCs, three GSC lines were cultured for 72 h in SFM supplemented with various PL concentrations: 1%, 2.5%, 5% or 10%. These media were compared to DMEM F12 SFM basic medium and DMEM-LG supplemented with 10% FCS as control media (Fig. 1A). GSCs cultured in SFM PL10% showed a significantly higher normalized proliferation rate (0.434 ± 0.006) compared to GSCs cultured in PL1% (0.306 ± 0.003), PL2.5% (0.355 ± 0.004) and PL5% (0.369 ± 0.005) (*p* < 0.005). GSCs cultured in DMEM F12 SFM basic medium showed no significant growth difference when compared to GSCs cultured in DMEM-LG supplemented with 10% FCS, which had the highest normalized proliferation rate overall (Fig. 1B and C).

To improve the GSC normalized proliferation rate in SFM PL10% medium, various growth factors were added to compensate for the absence of FCS 10%: GH, testosterone, EGF, TGFβ1 or bFGF (Fig. 1D). MTT assays of cells at 24 h and 72 h revealed higher cell proliferation in media supplemented with each factor (Fig. 1E). However, the results at 72 h showed that PL10% supplemented with GH at 4 ng/mL provided the highest growth (0.360 ± 0.015) when compared to SFM PL10% (0.322 ± 0.007) (Fig. 1E and F). Notably, the normalized proliferation rate was not significantly different between GSCs cultured in SFM PL10% and those cultured in SFM PL10% supplemented with any of the other factors (testosterone, EGF, TGFβ1 or bFGF) (Fig. 1F).

Cell-cycle analysis revealed that a large proportion of the GSCs cultured in SFM PL10% medium were in S-phase/Mitosis. This was also evident in SFM supplemented with GH, TGFβ1 or bFGF when compared to FCS10% medium, however these differences were not statistically significant (Fig. 1G).

#### SFM supplemented with PL 5% is optimal for mineralisation

We next aimed to validate the efficacy of SFM for osteogenic differentiation of GSCs. GSC and ASC lines were cultured for 21 days in various osteogenic media supplemented with different PL concentrations (1%, 2.5%, 5%, 10% or 20%) and compared to FCS osteogenic medium.

Cell viability was assessed by a Calcein AM assay and showed that SFM supplemented with PL1% and PL2.5% may be excluded, because the viability was below 50% for ASCs. The SFM supplemented with PL 5%, PL 10% and PL 20% supported viability levels ranging from 90 to 99% (Additional file 1: Figure S1A).

Spectrometric analysis of Alizarin Red S staining showed that for GSCs, SFM supplemented with PL2.5% and PL5% produced the best mineralisation rates when compared to SFM supplemented with PL1%, PL10% and PL20%, because of gel formation and contraction (in the highest concentrations, i.e., 10% and 20%). For ASCs, there was no significant difference in mineralisation rates amongst SFM supplemented with PL1%, PL2.5%, PL5% and PL10% (Additional file 1: Figure S1B).

Based on the optimal characterisation results from above, we used SFM supplemented with PL10% plus GH (4 ng/mL) for isolation and proliferation of GSCs, and SFM supplemented with PL5% for osteogenic differentiation, in all the following experiments.

### Isolation of GSCs in primary culture in SFM supplemented with PL10% + GH

**Fig. 2:**
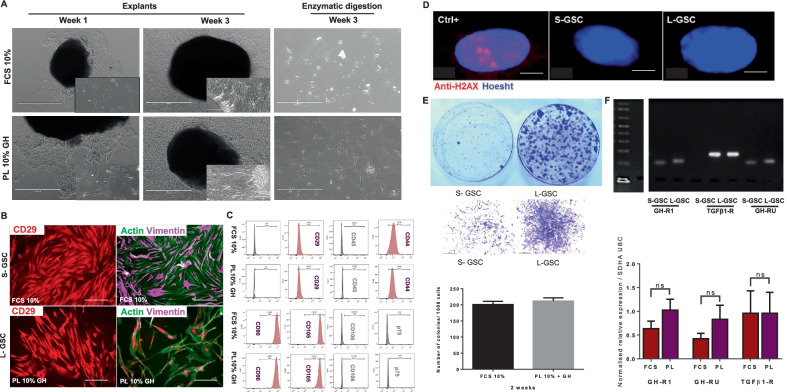
Isolation of GSCs in PL10% + GH medium and phenotypic properties. **A** Microscopic views of gingival fibroblasts (GFs) and GSCs isolated at 1 week and 3 weeks of explants and enzymatic digestion. GSCs retain their fibroblastic morphology in both FCS and PL media, but PL-isolated cells are larger. **B** CD29, actin and vimentin immunostaining of S-GSCs and L-GSCs. Cells were grown for 72 h on coverslips coated with gelatin 0.2%. **C** FACS analysis of MSC surface markers showed that both S-GSCs and L-GSCs were positive for CD29, CD44, CD90 and CD105. **D** DNA lesion detection was very low using Anti-H2AX staining in S-GSCs and L-GSCs. **E** and **F** Colony-forming unit-fibroblast (CFU-F) assay: Crystal violet staining showed that L-GSCs produce thicker and more robust colonies than S-GSCs, with a nearly similar number. (G and H) RT-qPCR gene expression analysis of GH-R1, GH-RU and TGFβ-R in both S-GSCs and L-GSCs. Gene expression was confirmed by migration of amplified DNA on an agarose gel, and the relative expression of the 3 genes was determined by normalisation with the *SDHA* and *UBC* as reference genes. No significant difference was found between S-GSCs and L-GSCs. Scale bars: **A** 1000 µM, 400 µM, **B** 400 µM, **D** 20 µM

Three lines of GSCs were successfully isolated in SFM supplemented with PL10% and GH (4 ng/mL) (PL10% + GH medium), either directly by the enzyme digestion method or by the explant technique, to first collect gingival fibroblasts (GFs), followed by limiting dilution (Fig. 2A). For the following experiments we used two types of GSCs: (1) L-GSCs harvested by limiting dilution, and (2) S-GSCs isolated in FCS10% medium.

After three weeks of primary culture, ~ 4 × 10^6^ GSCs were obtained in the two conditions (L-GSCs and S-GSCs) at passage 0 (P0) (i.e., before the first trypsin passage). L-GSCs retained their spindle-shaped morphology but were more likely to spread, giving larger fibroblast-like cells, and tended to form small colonies before reaching confluence (Fig. 2A).

Cell morphology was also similar, as shown by actin filament and vimentin immunofluorescence (Fig. 2B). Flow cytometry demonstrated that the vast majority of both L-GSCs and S-GSCs shared the same membrane markers: CD29, CD44, CD90 and CD105 (Fig. 2C).

H2AX immunofluorescence confirmed the absence of DNA lesions in both L-GSCs and S-GSCs, compared to positive control cells (Fig. 2D). Interestingly, the crystal violet staining showed that the use of PL10% + GH medium to isolate GSCs caused them to form larger and well-developed colonies when compared to S-GSCs (Fig. 2E), while the numbers of colonies were similar (Fig. 2F). RT-qPCR results confirmed the expression of the growth hormone receptor (GHR) and TGF-β receptor 1 in both L-GSCs and S-GSCs (Fig. 2G and H).

### Differentiation potency of primary L-GSCs cultured in SFM PL10% + GH

Simple differentiation methods were used to explore the multipotency of L-GSCs in comparison to S-GSCs. L-GSCs and S-GSCs were cultured in osteogenic, adipogenic, myofibroblastic and neurosphere-formation media.

**Fig. 3:**
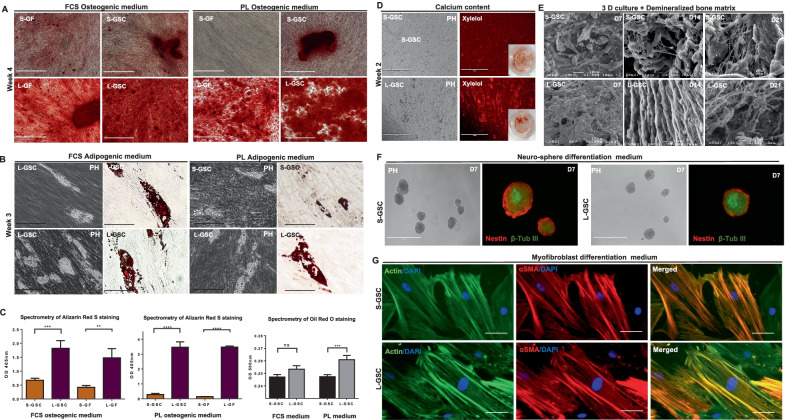
Osteogenic, adipogenic, myofibroblastic and neurosphere formation capacity of L-GSCs. **A** The osteogenic capacity was confirmed by Alizarin Red S staining after 28 days of culture of S-GFs, S-GSCs, L-GFs and L-GSCs in either FCS or PL osteogenic medium. **B** The adipogenic potential of L-GSCs and S-GSCs in both PL and FCS adipogenic media was confirmed by Oil Red O staining of the intracellular lipid vacuoles. **C** The spectrometry of Alizarin Red S staining showed that L-GFs and L-GSCs had 2–2.5-fold and fourfold higher values than S-GFs and S-GSCs in FCS and PL-osteogenic media, respectively. The spectrometry of Oil Red O staining showed no significant difference of the adipogenic capacity of L-GSCs and S-GSCs in FCS adipogenic medium, but a higher capacity of L-GSCs in PL-adipogenic medium. **D** Xylenol orange showed mineral deposits at day 15 of osteogenic differentiation in both S-GSCs and L-GSCs. **E** SEM imaging showed that both S-GSCs and L-GSCs were attracted to the allogenic Cancellous Particulate Allograft (ZIMMER-BIOMET) and formed mineral nodules around and between the particles of this matrix. **F** Immunostaining with Nestin and β3-tubulin antibodies confirmed the phenotype of neurospheres formed from S-GSCs and L-GSCs after 7 days of culture in untreated neurogenic medium. **G** Immunofluorescence labelling with α-SMA/Actin/Dapi antibodies in both groups of GSCs confirmed their capacity to undergo myofibroblastic differentiation after 5 days of culture under suitable conditions. Scale bars: **A** 1000 µM, **B** 100 µM, **D** 400 µM, **E** 400 µM, **F** 400 µM, **G** 25 µM

For the osteogenic differentiation experiment, both L-GSCs and S-GSCs, as well as L-GFs and S-GFs (non-CFU-F enriched) were cultured in osteogenic medium for 28 days, supplemented either with FCS20% or PL5% (Fig. 3A). PL-cultured GSCs and GFs underwent a faster and more robust osteogenic differentiation (4 weeks) compared to FCS-cultured cells, as highlighted by Alizarin Red S staining at day 28 (Fig. 3C) and xylenol orange staining at day 14 (Fig. 3D). In both media, L-GFs and L-GSCs showed more calcium content than did S-GFs and S-GSCs, which was confirmed by spectrometric values of Alizarin Red S staining of L-GSCs and L-GFs, which were fourfold those of S-GSCs and S-GFs, respectively (Fig. 3C). SEM imaging showed that both S-GSCs and L-GSCs could attach to a demineralized bone matrix (DBM), represented by an allogenic Cancellous Particulate Allograft (ZIMMER-BIOMET), and both cell types formed mineral nodules around and between the particles at days 7, 14 and 21 of osteogenic induction (Fig. 3E).

Both L-GSCs and S-GSCs differentiated after 21 days in adipogenic medium supplemented either with FCS10% or PL10% (Fig. 3B). Spectrometric analysis of Oil Red O staining revealed an increased content of lipid vacuoles for L-GSCs in both FCS and PL adipogenic media, but with a significantly higher value in PL adipogenic medium (Fig. 3C).

Further study of osteogenic and adipogenic differentiation was perfomed using RT-qPCR to analyse the expression of osteogenic (*ALP, DSPP, OCN and OPN*) and adipogenic markers (*PPAR γ* and *LPL*) in both S-GSCs and L-GSCs after 14 and 21 days of differentiation (Additional file 2: Figure S2A and B). Osteogenic markers were significantly increased in L-GSC as compared to S-GSC such as DSPP at day 14, ALP and OPN at day 21 of differentiation. In contrast, adipogenic markers were significantly more expressed for PPAR *γ* and LPL as compared to L-GSC.

For 3D culture, osteogenic differentiation potential of both S-GSC and L-GSC in a PL-gel supplemented with demineralized bone matrix (DBM) was studied. Alizarin red S and ALP staining after 21 days of differentiation. Cell viability at day 21 was confirmed by Calcein AM. RT-qPCR analysis for osteogenic markers like *ALP, DSPP and OCN* showed a significantly higher osteogenic potential of L-GSC in these conditions (Additional file 3: Figure S3A–D).

GSCs also formed neurospheres when cultured in untreated culture dishes with neurogenic medium. This was highlighted by the positive Nestin and β3-tubulin immunostaining in both S-GSCs and L-GSCs (Fig. 3F).

L-GSCs and S-GSCs underwent successful myofibroblastic differentiation after 5 days of culture in suitable conditions. This was revealed by the positive α-SMA immunofluorescence in both groups (Fig. 3G).

### Immunomodulatory properties of L-GSC

**Fig. 4:**
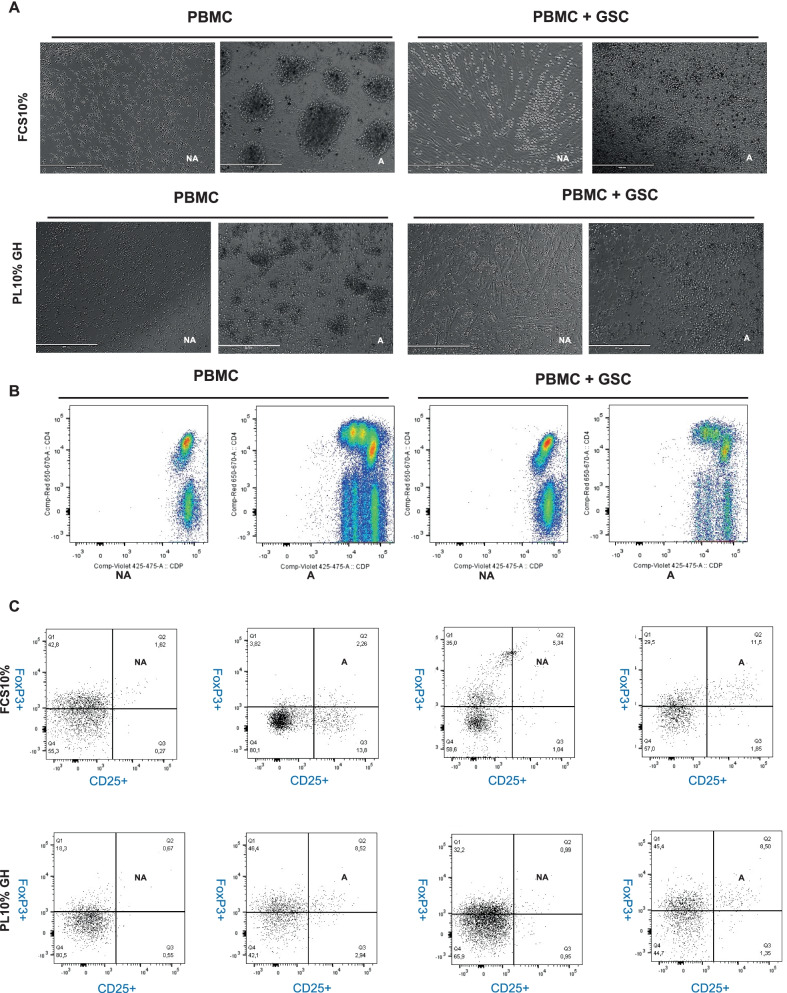
Immunomodulatory properties of L-GSCs. **A** Microscopic views of PBMCs after 72 h of culture under two different conditions: non-activated (NA) and activated (A), in the presence or absence of GSCs. PBMCs proliferated around Dyna-beads coupled to anti-CD3/CD28 (A condition), Scale bars: 400 µM. **B** FACS analysis of Cell Proliferation Dye (CPD) after 72 h of PBMC culture in the NA and A conditions, in the presence or the absence of GSCs. Cells proliferated in the A condition for 3 to 4 generations. **C** Flow cytometric analysis after 72 h of co-culture revealed that CD4+/CD3+/CD25+/FoxP3+ regulatory T cells were present in the A condition for both S-GSCs and L-GSCs

L-GSCs and S-GSCs were co-cultured with PBMCs in their respective proliferation media for 72 h, either in the presence (A condition) or in the absence (NA condition) of Anti-CD3/CD28 activation beads. Microscopic observations confirmed the activation of T cells in the “A” condition in both PL and FCS media by the attraction of these cells to, and their proliferation around, the beads. There was no proliferation of PBMCs in the “NA” condition, which confirms that 1) cell activation occurred only in the presence of activating beads and 2) L-GSCs display low immunogenicity, as previously described for S-GSCs (Fig. 4A). Flow cytometric analysis at 72 h by CDP fluorescence revealed no significant difference between the PBMC proliferation profiles in the “A” condition in the presence or absence of GSCs, albeit with a slight decrease of PBMC proliferation in the presence of GSCs (Fig. 4B).

CD3+/CD4+/ CD25+/FoxP3+ T cells were detected in the “A” condition after 72 h of co-culture in the presence of either S-GSCs or L-GSCs (Fig. 4C). CD4+ and CD8+ T-cell proliferation rates were similar in both FCS and PL media, with a slight but not significant decrease in PL medium. An examination of surface markers revealed no significant difference in proliferation rates at 72 h for CD4+ T-cell subpopulations (CD69+, CD25+, CD45RO+, CCR7, OX40, and ICOS), either in FCS or in PL media. For CD8+ subpopulations, there was a significant decrease of proliferation rate in PL medium for CD8+CD25+ and CD8+CD45RO+ subpopulations in the A condition in the absence of GSCs (Additional file 4: Figure S4).

## Discussion

Here we introduced a preclinical grade protocol for isolation and differentiation of oral GSCs in SFM supplemented with human PL. We have shown that the isolated GSCs retain their stemness potential, normalized proliferation rate and immunomodulatory properties normally observed when they are isolated in medium containing FCS.

The use of GSCs should be privileged in oral bone regeneration therapies for many reasons. For example, they are easy to access in the gingiva, and easily harvested with no scar formation [22]. Moreover, GSCs retain their stemness properties and display superior behaviour in vitro [17, 27], probably because they are most likely derived from cranial neural crest. The use of GSCs in craniofacial cell therapy represents a real alternative to the most commonly used cells, BM-MSCs [53] and AD-MSCs [20]. Although bone regeneration outcomes are relatively satisfactory for MSCs, differences in cellular phenotype and transcription profiles of these cells originating from bone marrow or adipose tissue may alter the long-term stability of the reconstructed sites and may explain the failure to ameliorate bone defects in some cases [28, 37, 45].

Our first goal was to isolate and proliferate GSCs in SFM at the same efficiency achieved with FCS-containing media. The use of PL was previously shown to increase the osteogenic and adipogenic differentiation potentials of GSCs [3, 10, 40], and we confirmed that efficacy here. It is interesting to highlight that PL 10%, which yielded the highest normalized proliferation rate of GSCs, needs be renewed in the culture medium every 3 days (data not shown). Indeed, a 7-day study would have introduced a bias linked to the renewal of the culture medium with new components. Nevertheless, the normalized proliferation rate for PL 10% was lower than that obtained with FCS 10%.

Similar observations were found with BM-MSCs cultured in PRP SFM (PPRF-msc6) [29]. It has also been reported that BM-MSCs exhibit a more elongated morphology [10, 48], as we reported for L-GSCs. However, both cell types expressed similar phenotypic markers. The PL medium supports cryopreservation of GSCs. Cryopreservation was possible in PL at − 80 °C for 3 months, beyond which the cells appear to have difficulty recovering (Additional file 5: Figure S5). Cryopreservation is of special importance since cell storage is needed for cell therapy and cell banking.

To have utility, SFM for stem cell culture must replace the contents of FCS that are essential for allowing the isolation and the differentiation of stem cells. Platelet-rich fibrins (PRFs) have been shown to induce osteoblastic differentiation of BM-MSCs [13]. Similarly, TGFβ1 contained in PL may increase the expression of Runx2 in the early stages of differentiation [5, 55]. In our study we supplemented our SFM with GH, which increased the osteoblastic differentiation of the GSCs. This may be explained as either a result of increased multipotency capacity of GSCs owing to Insulin Growth Factor I (IGF-1) contained in PL, or by a direct effect of GH on osteoblastic differentiation through GHR. During bone remodelling, IGF-1 is released into the bone matrix to stimulate differentiation of MSCs into osteoblasts by activation of the mTOR receptor [52]. The combination of PDGF and IGF-1 has been shown to be more effective than PDGF alone in terms of osteogenic induction of AD-MSCs . Cells cultured on porous allogeneic bone matrix in 3D showed good viability (data not shown) and we observed GSC mineralisation around and between the nodules of this matrix for both L-GSCs and S-GSCs. GH has also been shown to play an important role in increasing the osteogenic potential of 3D-grown MSCs on nanoparticles [49]. In our study, the normalized proliferation rate was improved in the presence of EGF, TGFβ1, GH and testosterone separately. Cells in the presence of GH with PL10% showed significantly higher normalized proliferation rates, and this was confirmed by flow cytometry results at 24 h (data not shown), showing an increased proportion of cells in S phase in this new medium.

GH is widely used in endocrinology. It was demonstrated to have a positive effect on wound healing [30]. However, GH is very rarely used for amplification of MSCs, although at least one in vitro study showed that a higher rate of MSC proliferation could be obtained by adding GH (10 μg/L) to the culture medium [38]. In our study, we kept the same concentration of GH as approximately found in FCS (4 μg/L) and to be comparable with the control medium (FCS10%). Moreover, PL provides a very good source for the multiple growth factors necessary for proliferation, particularly IGF-1, by which GH acts secondarily at the MSC level. Molecular studies of GH have reported that GH acts via IGF-I in wound healing when applied locally [41], and it increases fibroblast proliferation and migration of keratinocytes either directly on cell progenitors or indirectly by significantly increasing cell proliferation via IGF-1 [36]. In addition, under culture conditions without serum, MSCs were found to proliferate in response to IGF-1 [42], which produces its effect by inducing several intracellular signalling pathways [32]. Although the effects of GH are mainly explained by its increased synthesis of IGF-1, its mechanism of direct action on proliferation remains poorly described and requires further studies to confirm its mechanism of action. Moreover, GH Receptor 1 (GHR1) and the universal GH receptors (GHRs) were positively expressed in both S-GSCs and L-GSCs. This may be attributed to the presence of GH in both FCS and our new SFM.

The immunomodulatory role of MSCs has been thoroughly reported and investigated [12, 34]. This property has been highlighted for GSCs [7, 54]. To better test the conservation of the immunomodulatory properties of GSCs in this new SFM, we examined their effect on the proliferation of antigen-stimulated T cells. The proliferation of regulatory T cells was increased in the presence of S-GSCs and L-GSCs. This confirmed that GSCs in our new SFM act on the lymphocyte profile by increasing the CD4+ regulatory population and decreasing the populations of regulatory cytotoxic and memory T cells. This could be explained by the presence of platelet growth factors that may play a role in decreasing these cell populations. CD8+/CD25+ cells are induced by viral infection and some mechanisms dependent on IL-4 and IL-12, and they regulate Th1/Th2 reactions by production of IL-10 and IFNγ [1, 44]. More studies are needed to confirm this trend, including investigation of cytokines and their actions on natural killer cells, M2 macrophages and dendritic cells. Recently, it was shown that culturing and isolation of BM-MSCs or AD-MSCs in PL-supplemented medium does not change their immunomodulatory properties [1]. We confirmed these results on GSCs by analysis of the lymphocyte profile through activated PBMCs in vitro. We observed preservation of the lymphocyte profile of the CD4+ subpopulations in FCS10% and PL10% + GH media in the presence or absence of GSCs. On the other hand, when used in cell therapy, the immunomodulatory properties of GSCs could be exploited to reduce bone graft adverse reactions, especially for allogeneic grafts. This may be explained by the maintenance of immune balance by L-GSCs during inflammatory reactions. Their absence leads to a significant reduction in the proliferation of CD8+ subpopulations, namely CD45RO+ and CD25. Finally, the numbers of cytotoxic memory and regulatory cells are significantly increased in the presence of L-GSCs, which shows conservation of the role of GSCs in maintaining immune balance in the lymphocyte population during an inflammatory reaction.

## Conclusion

The aim of this study was to validate a defined SFM and cell-culture protocol adapted to GSCs, starting from GSC isolation from gingival tissue and demonstrating their osteogenic differentiation, ultimately for the purpose of treating large maxillary bone defects in accordance with good manufacturing practice (GMP) guidelines and compatible with human cell therapy. The SFM supplemented with 10% PL and GH sustained the normalized proliferation rate of GSCs. The mineralisation was efficient when GSCs were isolated in this new SFM. The phenotypic and immunomodulatory properties were conserved. We confirm the potential use of these new culture conditions for GSCs for human cell-based therapies.

## Supplementary Information

**Additional file 1: Figure S1**. Validation of PL5% as the ideal concentration for GSC osteogenic differentiation medium. (A) Calcein AM/Dapi viability assay showed that the SFM osteogenic conditions PL 5%, 10% and 20% had a viability rate ranging between 90 and 99% for both GSCs and adipose-derived stem cells (ASCs). (B) The spectrometry of Alizarin Red S staining showed that PL 2.5% and PL 5% had the highest increase for nodule mineralisation in GSCs.

**Additional file 2: Figure S2**. Expression of osteogenic and adipogenic markers of S-GSC and L-GSC. RT-qPCR analysis for (A) osteogenic (*ALP*, *DSPP*, *OCN* and *OPN*) and (B) adipogenic markers (*PPAR* *γ* and *LPL*) and in both S-GSCs and L-GSCs after 14 and 21 days of differentiation. Datas were normalized with *SDHA* and *GAPDH* as reference genes for osteogenic differentiation and *GAPDH* for adipogenic differentiation. One-way ANOVA test was performed for statistical analysis (p<0.05).

**Additional file 3: Figure S3**. 3D culture of GSC with demineralized bone matrix. (A) Osteogenic differentiation potential of both S-GSC and L-GSC in a PL-gel supplemented with demineralized bone matrix (DBM) for was confirmed by Alizarin red staining S after 21 days of differentiation. (B) Cell viability at day 21 was confirmed by Calcein AM. (C) ALP staining of S-GSC and L-GSC in a PL-gel was positive. (D) RT-qPCR analysis for osteogenic markers (*ALP*, *DSPP*, *OCN* and *OPN*) showed a significantly higher osteogenic potential of L-GSC in these conditions.

**Additional file 4: Figure S4**. Effects of PBMC coculture with L-GSCs and S-GSCs on T-cell phenotypes. (A, B and C) Flow cytometry analysis of Cell Proliferation Dye (CPD) staining in activated T cells after PBMC culture for 72 h in the presence of Dyna-beads anti CD3/CD28. T-cell surface marker analysis showed similar proliferation rates for CD4+ T cells in all FCS and PL conditions, but a significant decrease of CD8+ CD45RO+ and CD8+/CD25+ in the S condition in PL medium. NA: non-activated, A: Activated.

**Additional file 5: Figure S5**. Cryopreservation and maintenance of the osteogenic potential of L-GSCs post-thawing. (A, B and C) Thawed GSCs previously cryopreserved in a mixture of PL 50%/PL+GH SFM 40%/DMSO10% for 3 months at −80 °C. Cells retained their osteogenic and adipogenic capacities and growth rate.

## Data Availability

Raw data are available upon reasonable request to the corresponding author.

## Abbreviations

GSC: Gingival stem cell
FCS: Foetal calf serum
SFM: Serum-free medium
PBMC: Peripheral blood monocellular cell
MSC: Mesenchymal stem cell
GMP: Good manufacturing practice
AD-MSC: Adipose-derived MSC
DMSO: Dimethylsulfoxide
DBM: Demineralized bone matrix
