# Supplementary figures and images for: Efficient isolation of human gingival stem cells in a new serum-free medium supplemented with platelet lysate and growth hormone for osteogenic differentiation enhancement
Source: Stem Cell Res Ther. 2022 Mar 25;13:125. doi: 10.1186/s13287-022-02790-7 (PMC8951723; doi:10.1186/s13287-022-02790-7)

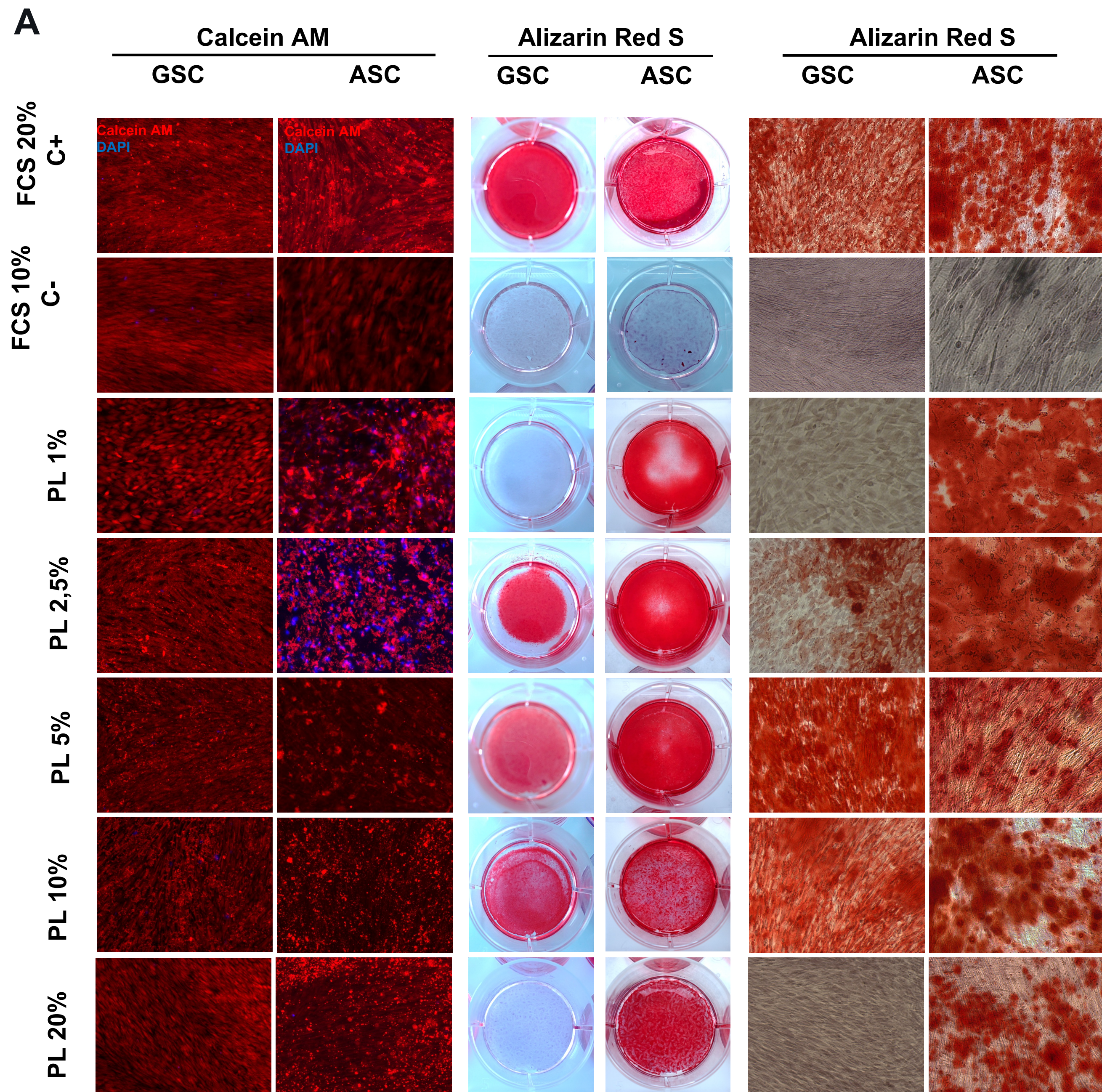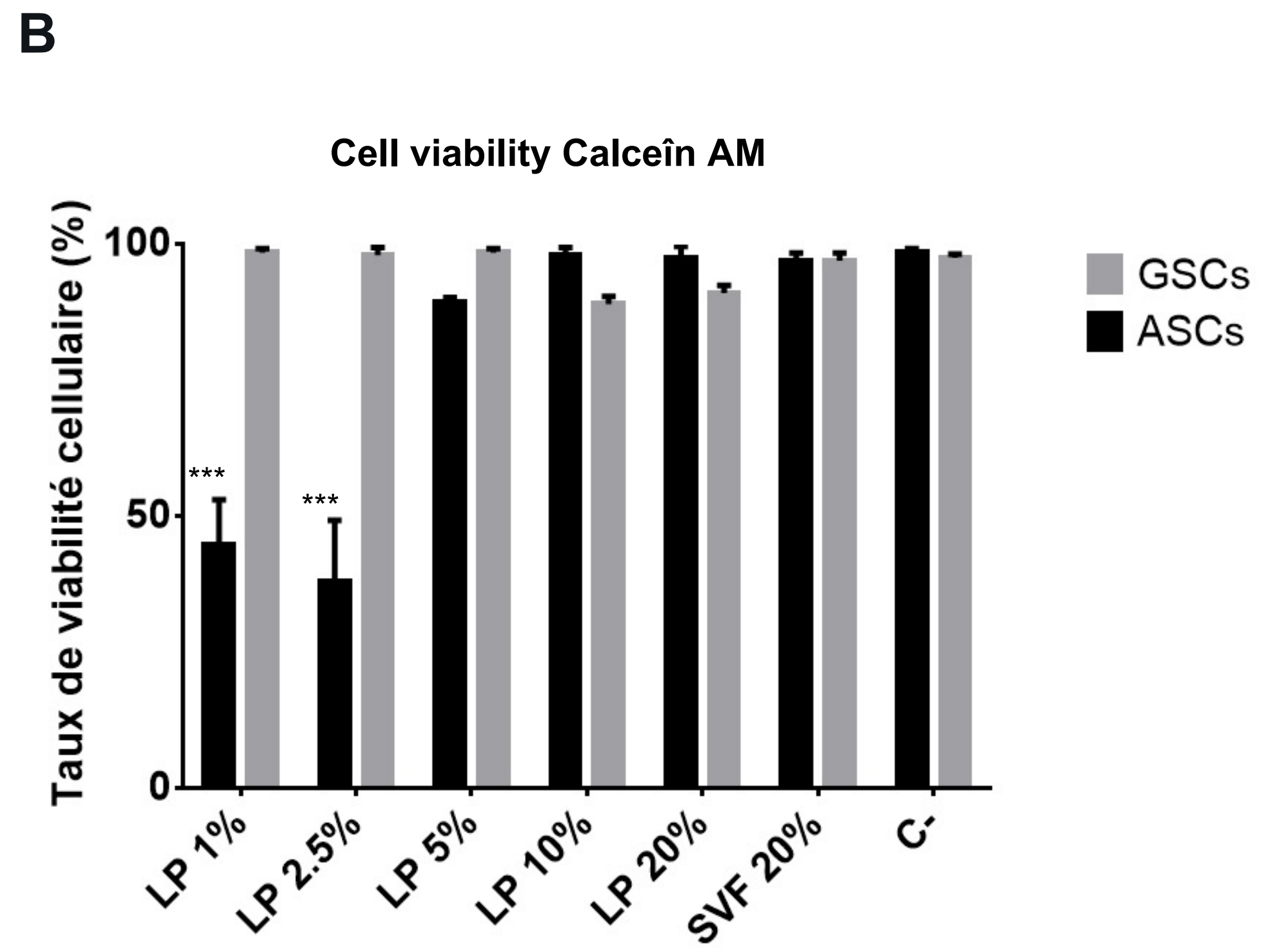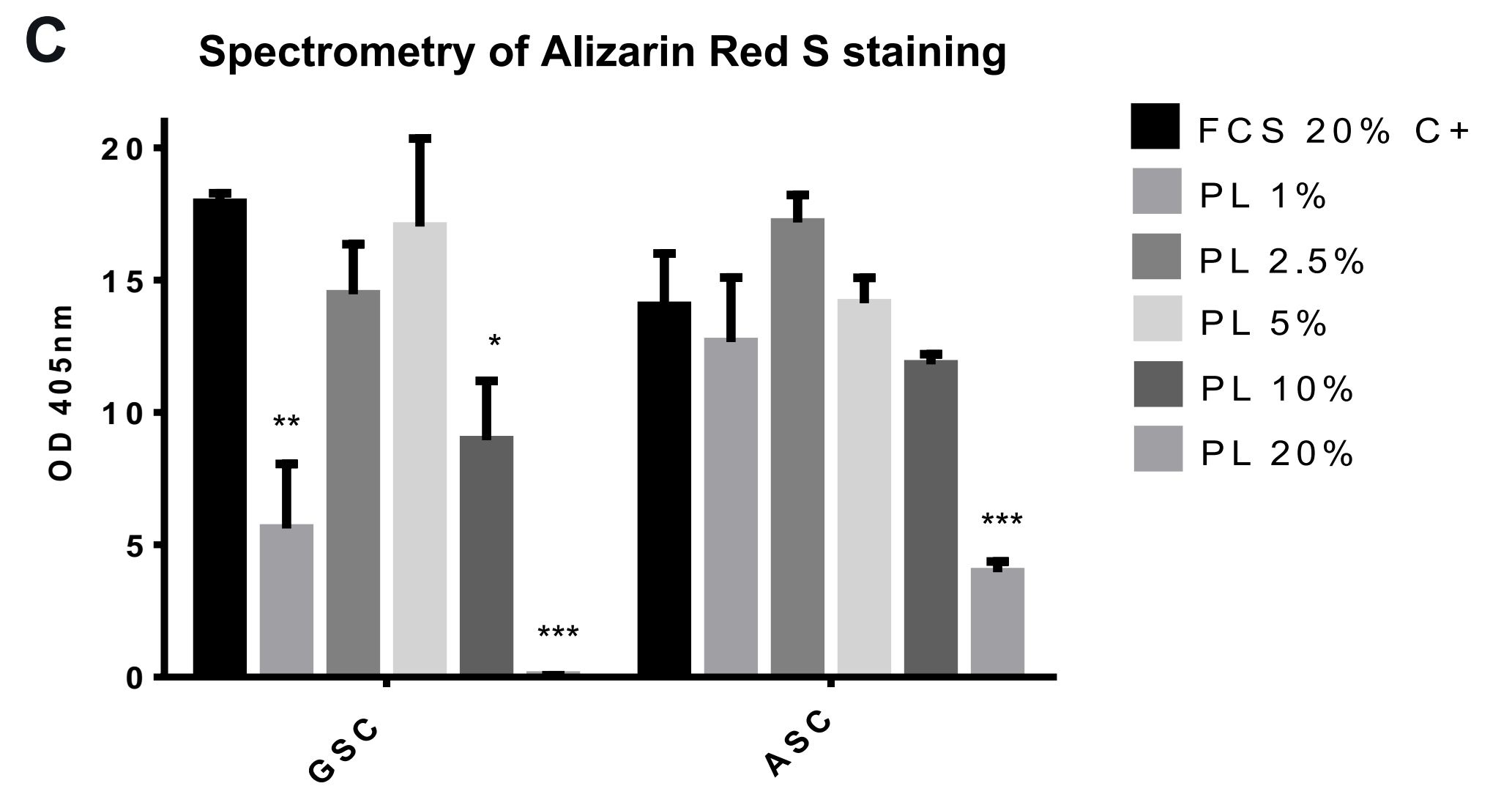

Supplement: Supplementary file 1 — Additional file 1: Figure S1. Validation of PL5% as the ideal concentration for GSC osteogenic differentiation medium. (A) Calcein AM/Dapi viability assay showed that the SFM osteogenic conditions PL 5%, 10% and 20% had a viability rate ranging between 90 and 99% for both GSCs and adipose-derived stem cells (ASCs). (B) The spectrometry of Alizarin Red S staining showed that PL 2.5% and PL 5% had the highest increase for nodule mineralisation in GSCs. [file 13287_2022_2790_MOESM1_ESM.pdf]

## Osteogenic differentiation

**A**

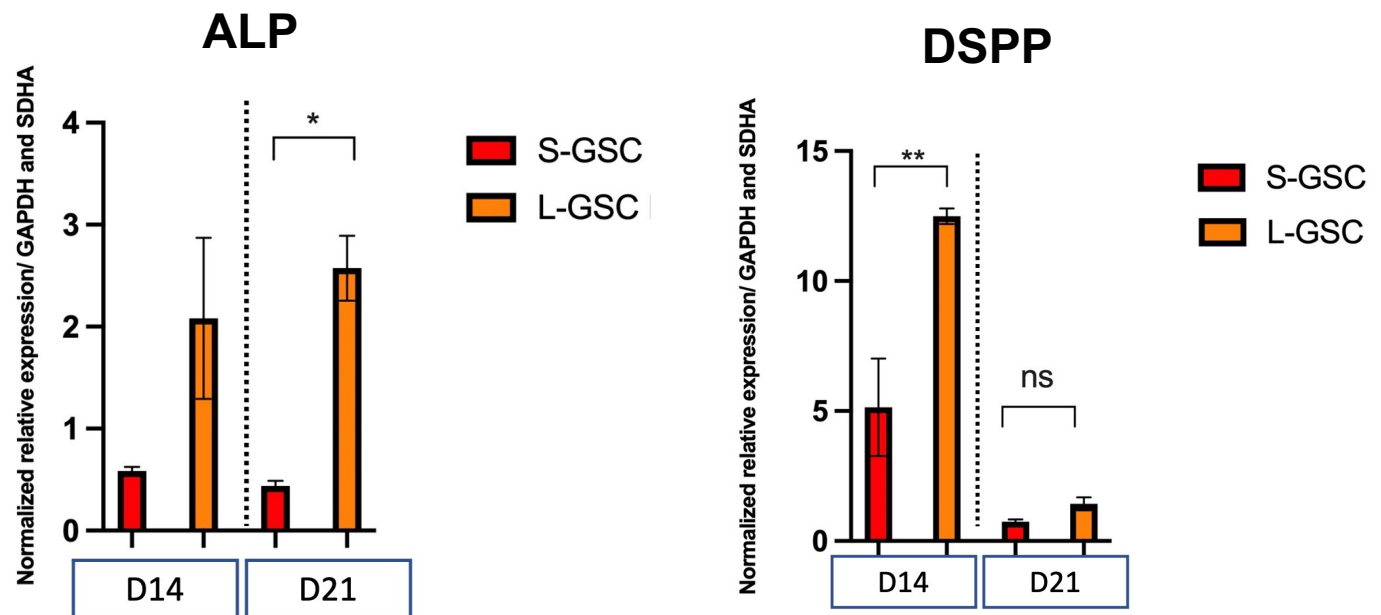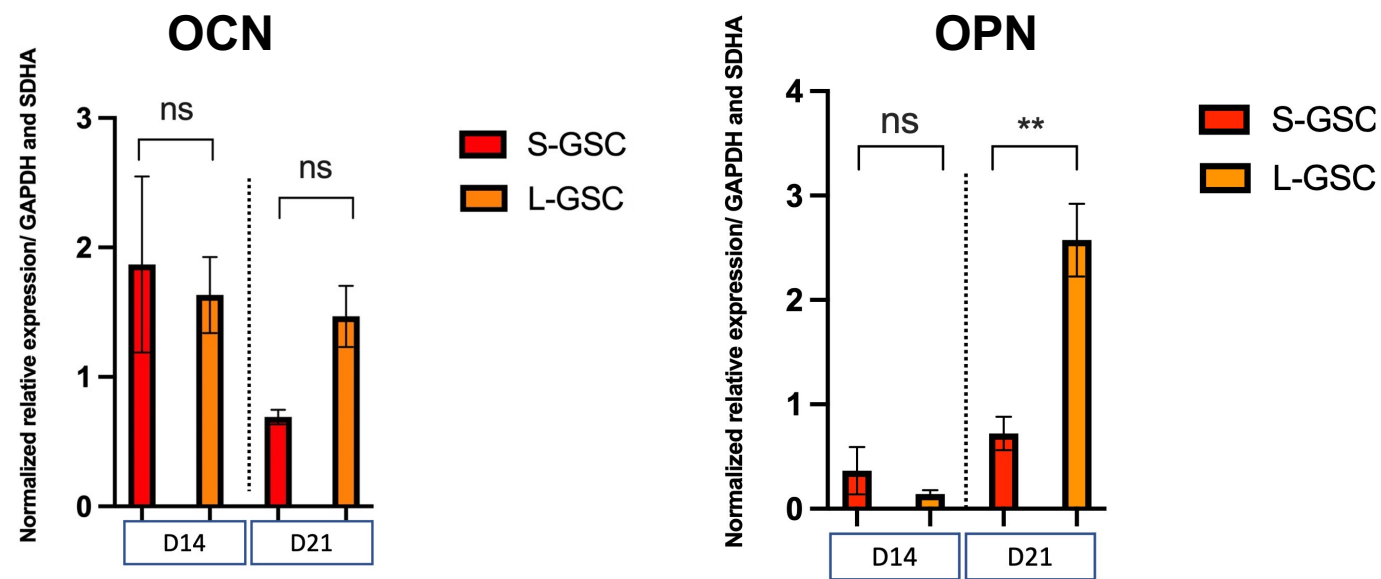

## Adipogenic differentiation

**B**

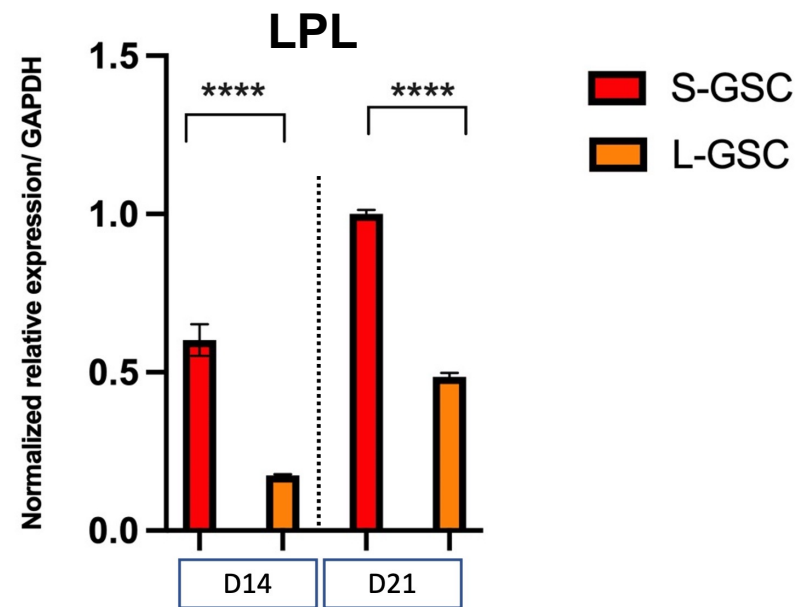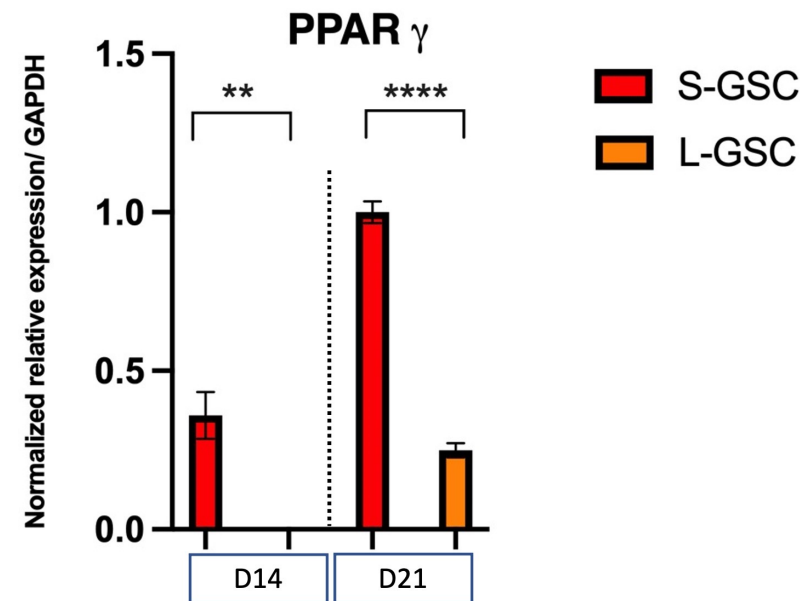

Supplement: Supplementary file 2 — Additional file 2: Figure S2. Expression of osteogenic and adipogenic markers of S-GSC and L-GSC. RT-qPCR analysis for (A) osteogenic (ALP, DSPP, OCN and OPN) and (B) adipogenic markers (PPAR γ and LPL) and in both S-GSCs and L-GSCs after 14 and 21 days of differentiation. Datas were normalized with SDHA and GAPDH as reference genes for osteogenic differentiation and GAPDH for adipogenic differentiation. One-way ANOVA test was performed for statistical analysis (p<0.05). [file 13287_2022_2790_MOESM2_ESM.pdf]

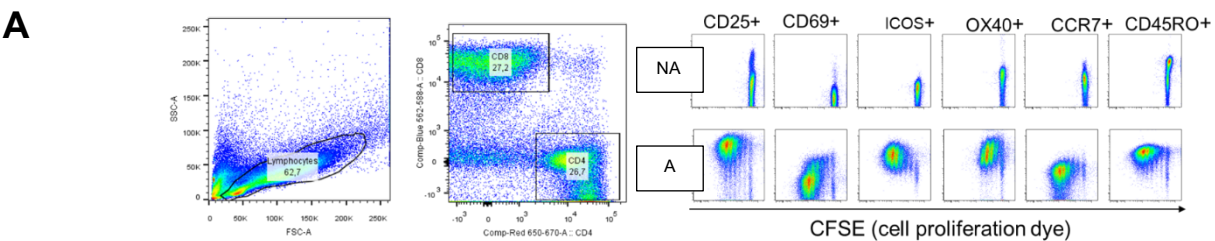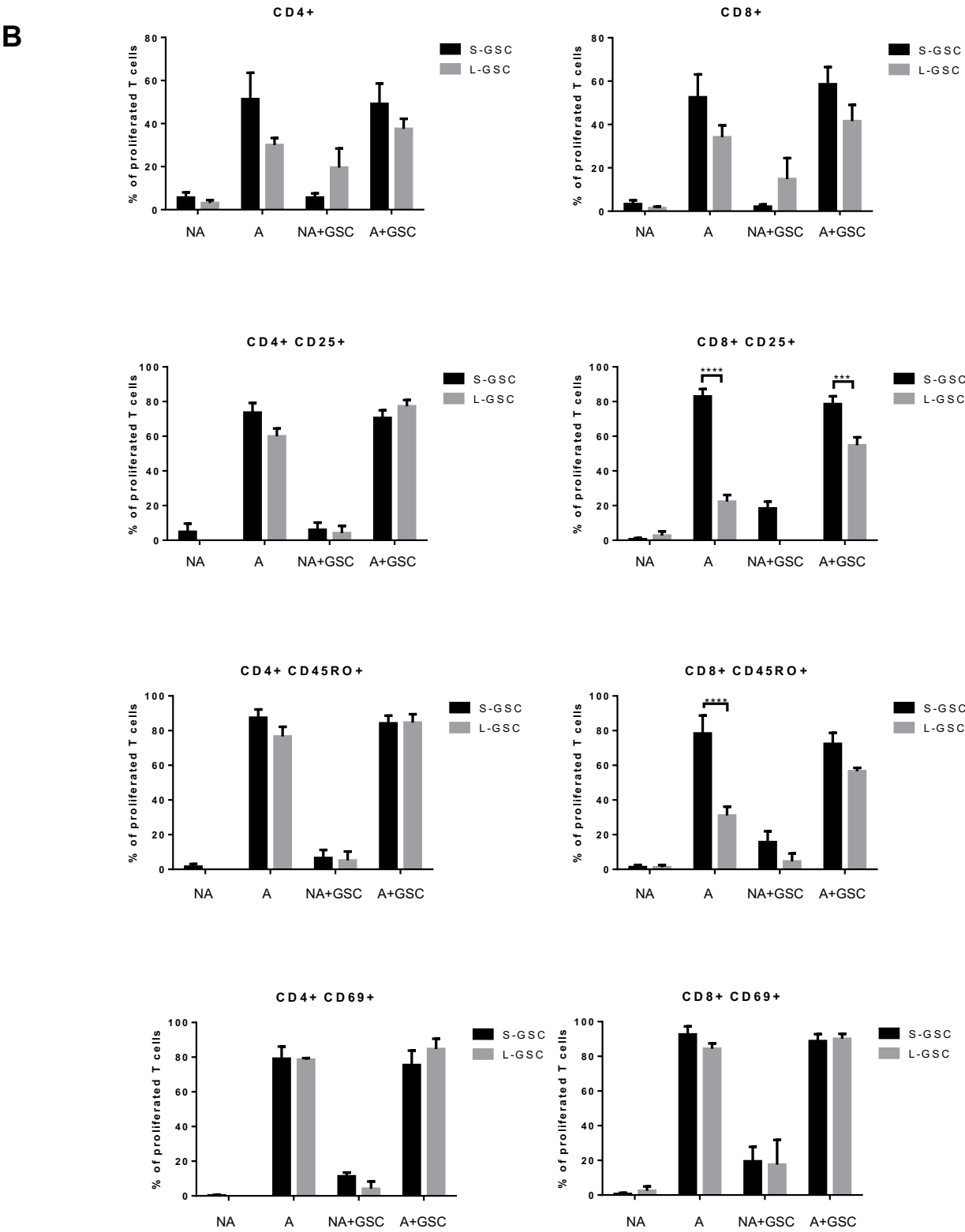

C

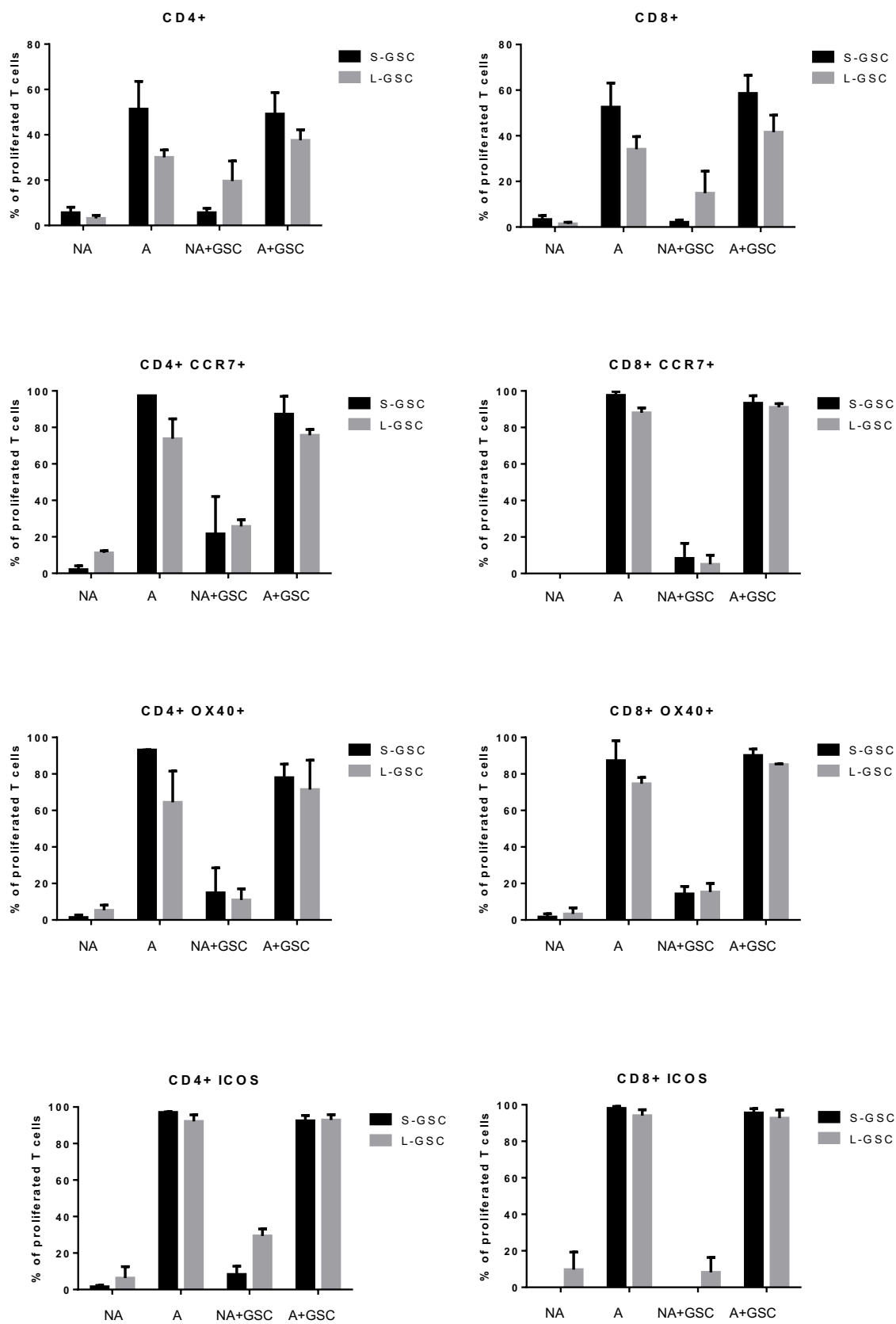

Supplement: Supplementary file 4 — Additional file 4: Figure S4. Effects of PBMC coculture with L-GSCs and S-GSCs on T-cell phenotypes. (A, B and C) Flow cytometry analysis of Cell Proliferation Dye (CPD) staining in activated T cells after PBMC culture for 72 h in the presence of Dyna-beads anti CD3/CD28. T-cell surface marker analysis showed similar proliferation rates for CD4+ T cells in all FCS and PL conditions, but a significant decrease of CD8+ CD45RO+ and CD8+/CD25+ in the S condition in PL medium. NA: non-activated, A: Activated. [file 13287_2022_2790_MOESM4_ESM.pdf]

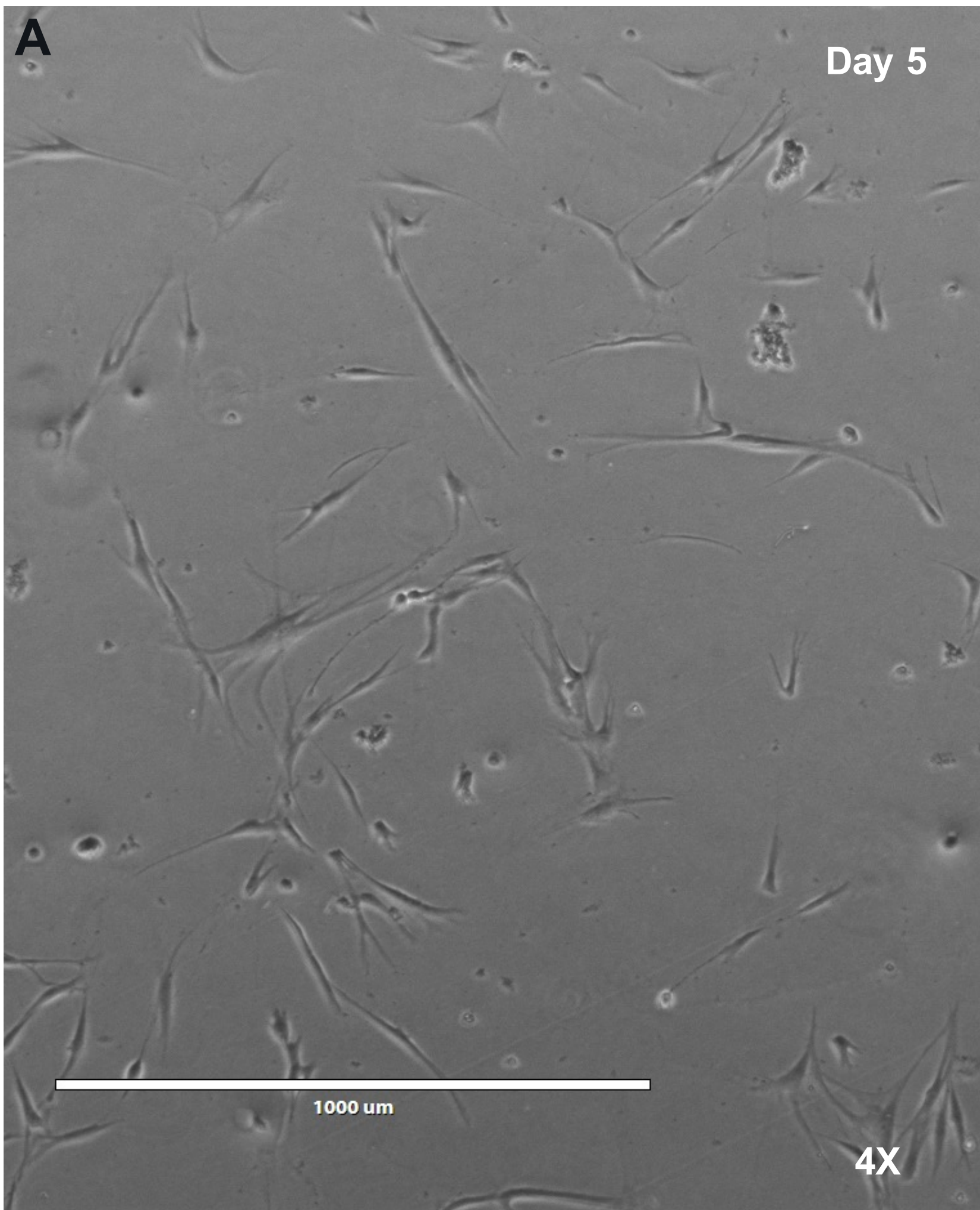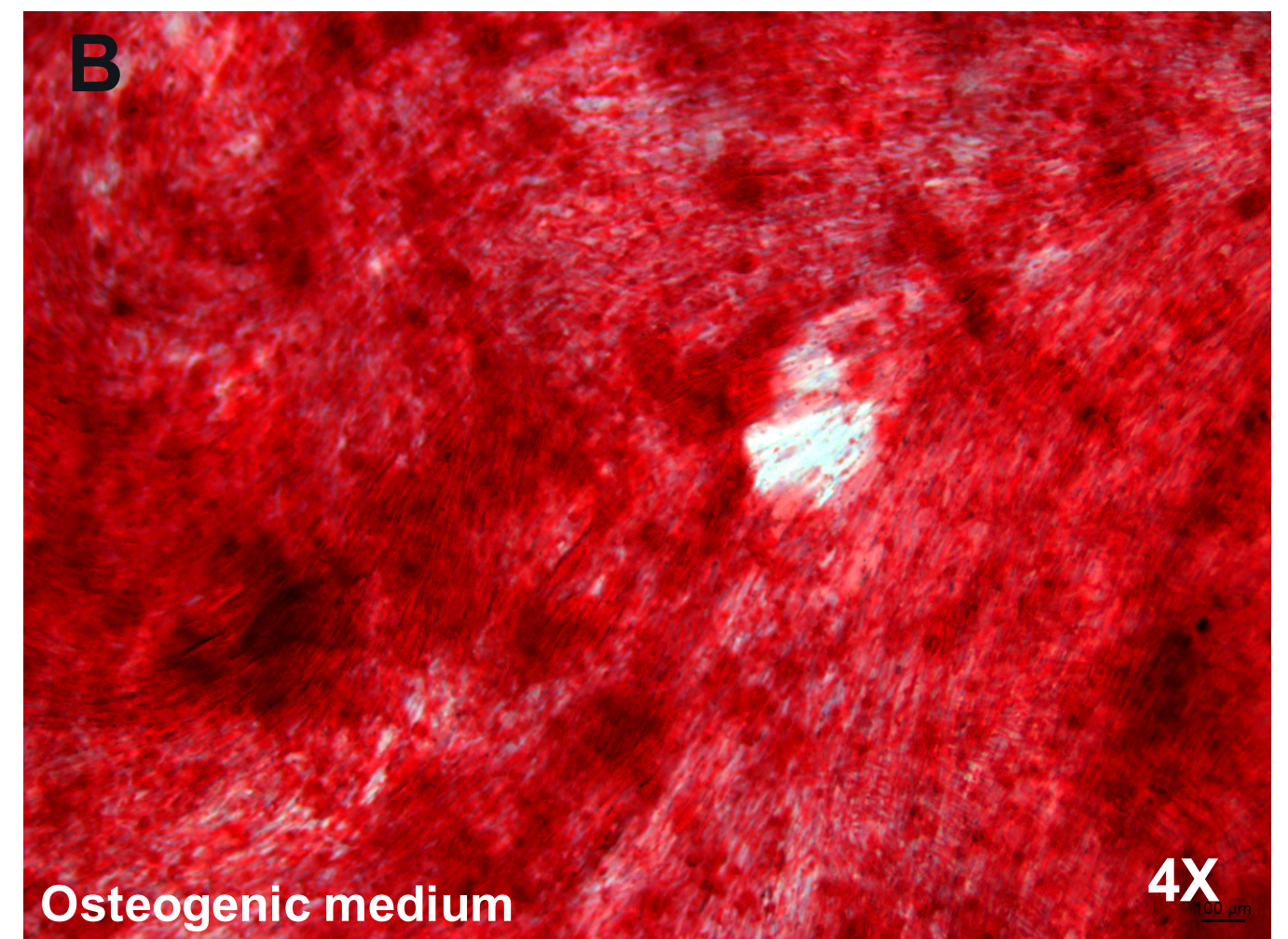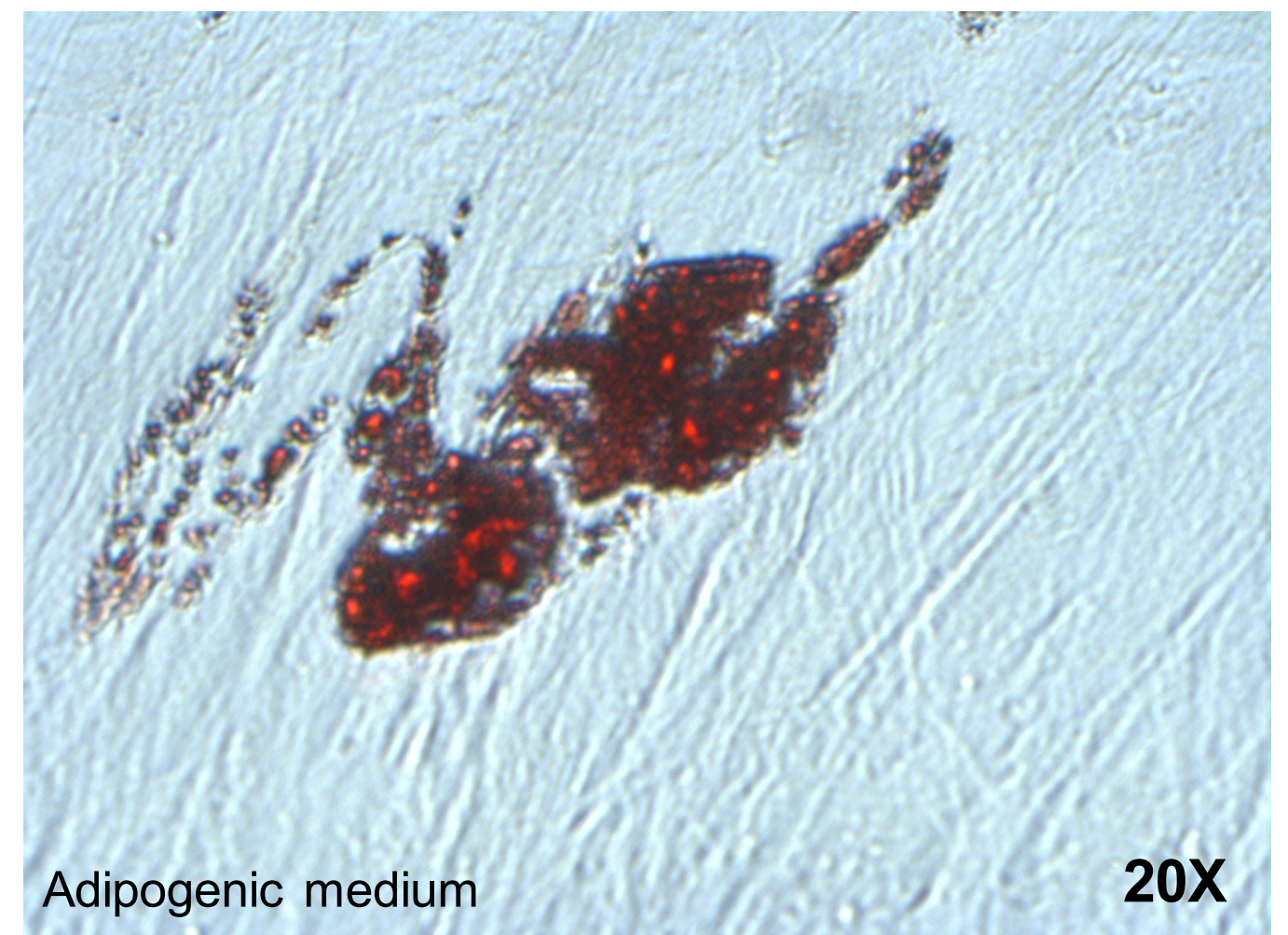

Supplement: Supplementary file 5 — Additional file 5: Figure S5. Cryopreservation and maintenance of the osteogenic potential of L-GSCs post-thawing. (A, B and C) Thawed GSCs previously cryopreserved in a mixture of PL 50%/PL+GH SFM 40%/DMSO10% for 3 months at −80 °C. Cells retained their osteogenic and adipogenic capacities and growth rate. [file 13287_2022_2790_MOESM5_ESM.pdf]
