# Supplementary material for: Efficient isolation of human gingival stem cells in a new serum-free medium supplemented with platelet lysate and growth hormone for osteogenic differentiation enhancement
Source: Stem Cell Res Ther. 2022 Mar 25;13:125. doi: 10.1186/s13287-022-02790-7 (PMC8951723; doi:10.1186/s13287-022-02790-7)
Supplement: Supplementary file 3 — Additional file 3: Figure S3. 3D culture of GSC with demineralized bone matrix. (A) Osteogenic differentiation potential of both S-GSC and L-GSC in a PL-gel supplemented with demineralized bone matrix (DBM) for was confirmed by Alizarin red staining S after 21 days of differentiation. (B) Cell viability at day 21 was confirmed by Calcein AM. (C) ALP staining of S-GSC and L-GSC in a PL-gel was positive. (D) RT-qPCR analysis for osteogenic markers (ALP, DSPP, OCN and OPN) showed a significantly higher osteogenic potential of L-GSC in these conditions. [file 13287_2022_2790_MOESM3_ESM.pdf]

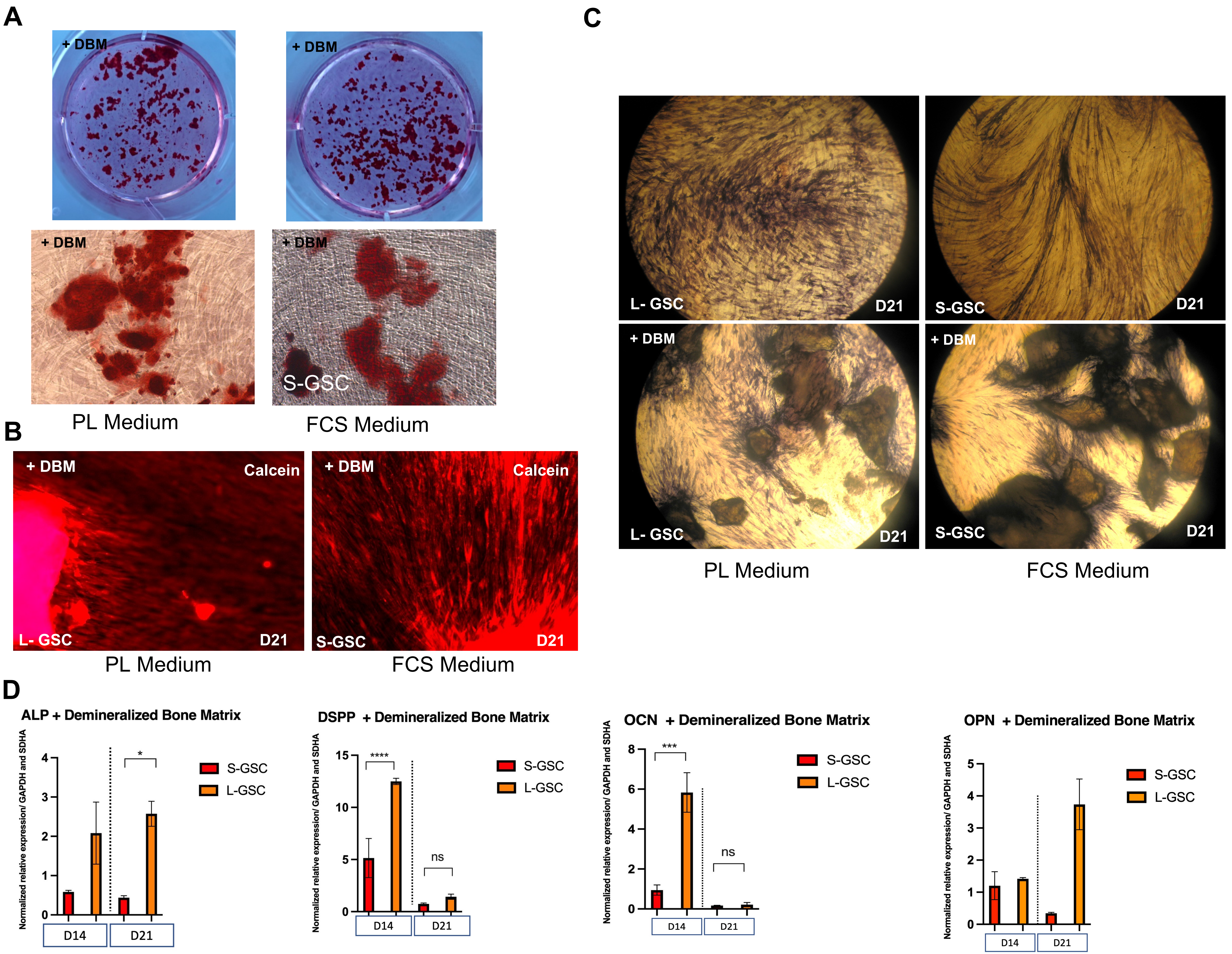
A
+ DBM
+ DBM
+ DBM
+ DBM
S-GSC
PL Medium
FCS Medium
B
+ DBM
Calcein
L- GSC
D21
+ DBM
Calcein
S-GSC
D21
PL Medium
FCS Medium
C
L- GSC
D21
S-GSC
D21
+ DBM
L- GSC
D21
+ DBM
S-GSC
D21
PL Medium
FCS Medium
D
ALP + Demineralized Bone Matrix
DSPP + Demineralized Bone Matrix
OCN + Demineralized Bone Matrix
OPN + Demineralized Bone Matrix
Normalized relative expression/ GAPDH and SDHA
S-GSC
L-GSC
D14
D21
*
****
***
ns
